# Modeling Musculoskeletal Disorders in Zebrafish: Advancements in Muscle and Bone Research

**DOI:** 10.3390/cells14010028

**Published:** 2024-12-30

**Authors:** Luca Dalle Carbonare, Michele Braggio, Arianna Minoia, Mattia Cominacini, Maria Grazia Romanelli, João Pessoa, Natascia Tiso, Maria Teresa Valenti

**Affiliations:** 1 Department of Engineering for the Innovation Medicine, University of Verona, 37100 Verona, Italy; luca.dallecarbonare@univr.it (L.D.C.); arianna.minoia@univr.it (A.M.); mattia.cominacini@univr.it (M.C.); 2 Department of Neurosciences, Biomedicine and Movement Sciences, University of Verona, 37100 Verona, Italy; michele.braggio@univr.it (M.B.); mariagrazia.romanelli@univr.it (M.G.R.); 3 Department of Medical Sciences and Institute of Biomedicine—iBiMED, University of Aveiro, 3810-193 Aveiro, Portugal; joao.pessoa@ua.pt; 4 Department of Biology, University of Padua, 35131 Padua, Italy; natascia.tiso@unipd.it

**Keywords:** zebrafish, bone, musculoskeletal disorders, aging

## Abstract

Zebrafish (*Danio rerio*) have emerged as a valuable model organism for investigating musculoskeletal development and the pathophysiology of associated diseases. Key genes and biological processes in zebrafish that closely mirror those in humans, rapid development, and transparent embryos make zebrafish ideal for the in vivo studies of bone and muscle formation, as well as the molecular mechanisms underlying musculoskeletal disorders. This review focuses on the utility of zebrafish in modeling various musculoskeletal conditions, with an emphasis on bone diseases such as osteoporosis and osteogenesis imperfecta, as well as muscle disorders like Duchenne muscular dystrophy. These models have provided significant insights into the molecular pathways involved in these diseases, helping to identify the key genetic and biochemical factors that contribute to their progression. These findings have also advanced our understanding of disease mechanisms and facilitated the development of potential therapeutic strategies for musculoskeletal disorders.

## 1. Introduction

The zebrafish (*Danio rerio*) is a member of the largest and most diverse group of bony fish known, Teleostei. Teleosts account for the majority of modern fish species and are characterized by features such as a mobile jaw, symmetrical tails, and lightweight, flexible scales, all of which have contributed to their evolutionary success [1]. Like other teleosts, zebrafish possess an endoskeleton inherited from a common ancestor shared with mammals [1]. This evolutionary association provides important similarities in skeletal development, making zebrafish a valuable model for studying bone biology and the related genetic mechanisms. Indeed, the zebrafish has gained prominence as a model organism in developmental biology for several reasons, primarily its genetic and physiological similarities to humans [2]. Approximately 70% of human genes have functional orthologues in zebrafish, and many of these genes are associated with human diseases, making zebrafish an excellent system for studying a wide range of biological processes, including those involved in human pathologies [3,4]. Zebrafish embryos are transparent, and this characteristic allows for the real-time and non-invasive imaging of developing tissues and organs, including bone and muscle, from the earliest stages of development [5,6,7]. Additionally, the external fertilization and rapid development in zebrafish allow one to observe the crucial developmental stages within days, significantly accelerating the data acquisition compared to other animal models like mice [8,9,10]. The zebrafish skeletal system undergoes ossification through both intramembranous and endochondral pathways, reflecting the bone formation processes observed in mammals [11,12]. Similarly, zebrafish skeletal muscle development shares key regulatory pathways with humans, making this model particularly suited for studying musculoskeletal development and the related disorders [12].

Bone–muscle interaction occurs through multiple mechanisms, including mechanical forces generated by muscle contractions that directly stimulate bone formation, as well as biochemical signaling pathways involving molecules such as myokines (muscle-derived signaling proteins) and osteokines (bone-derived signaling factors) [13,14,15,16]. These molecules help coordinate the adaptive responses in bone and muscle in relation to physical activity or injury [17,18,19]. Mechanical loading during muscle contraction stimulates osteoblast activity, promoting bone growth and strengthening [20,21,22]. Conversely, bone-secreted factors influence muscle function and regeneration. This relationship is particularly important in understanding musculoskeletal disorders, where dysfunction in one tissue often negatively impacts the other. For instance, in diseases like osteoporosis and muscular dystrophy, the integrity of both muscle and bone is compromised, leading to progressive degeneration and reduced functional capacity [23,24,25]. Zebrafish models allow researchers to dissect these interactions in real time, providing key insights into the molecular mechanisms driving bone and muscle diseases and identifying the potential therapeutic targets aimed at restoring or enhancing muscle–bone communication. However, there are still relatively few studies on bone–muscle crosstalk, highlighting the need for further research to fully understand the mechanisms underlying this interaction [26].

## 2. Zebrafish as a Model for Musculoskeletal Disorders

The zebrafish is an invaluable model for studying both muscle and bone, offering a dynamic platform to explore the genetic and environmental factors contributing to musculoskeletal pathologies. Musculoskeletal disorders, such as muscular dystrophies and osteopathies, lead to severe disability [27,28]. Zebrafish offer a unique opportunity to model such diseases due to their genetic tractability and the ability to perform large-scale mutagenesis screens and CRISPR-Cas9 gene editing [29,30,31]. Moreover, the external development and transparency of zebrafish allow researchers to perform real-time fluorescence imaging during skeletal development. Indeed, zebrafish transparency enables the in vivo tracking of bone cell differentiation using transgenic lines expressing fluorescent markers [32]. The transparency in the early developmental stages supports the live imaging of organ formation, while advanced imaging techniques extend the study of musculoskeletal processes into adulthood [32]. The key protein domains involved in chain recognition and protein binding are highly conserved between zebrafish and humans. Zebrafish bone cells, gene expression, and remodeling processes resemble those of mammals, allowing insights into bone turnover [33].

Additionally, zebrafish offer a robust toolkit for rapid and precise genome editing, enhancing their utility in modeling complex diseases. Collaborative efforts within the research community have contributed to the sharing of mutant lines and the associated phenotypic data. The Zebrafish Model Organism Database (ZFIN; http://zfin.org) is a centralized repository platform for the exchange of genetic, genomic, and phenotypic information [34]. Additionally, resources such as the Zebrafish International Resource Center (ZIRC) and the European Zebrafish Resource Center (EZRC) provide access to zebrafish strains, including wildtype and mutant, and reagents such as zebrafish-specific antibodies [33,35]. Several studies have used zebrafish models to identify the key molecular pathways in musculoskeletal diseases, highlighting the potential therapeutic targets.

### 2.1. Bone Disorders

**Figure 1:**
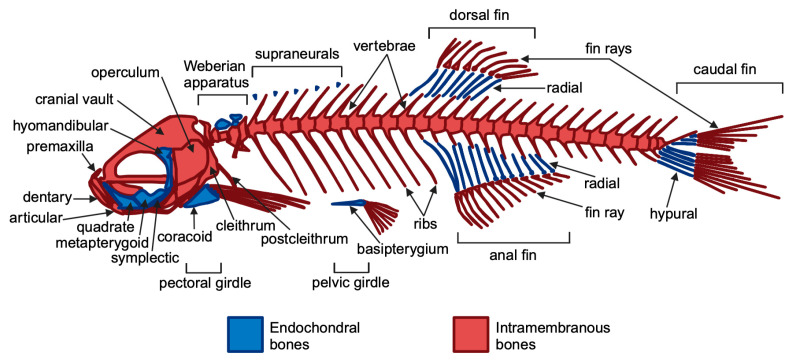
General overview of the zebrafish adult skeleton. Endochondral and intramembranous bones are indicated in blue and red, respectively. Figure created using BioRender.com and adapted from Le Pabic et al., 2022 [41].

Bone is a unique tissue in vertebrates, providing internal structural support [36]. Across vertebrate species, skeletal structures, such as the skull, vertebrae, and appendages, share remarkable similarities. Fossil studies reveal that these structures originated from homologous bones, which have been conserved and modified throughout evolution in fish, amphibians, reptiles, and mammals [37]. These evolutionary relationships highlight the shared developmental and genetic pathways governing bone formation, making vertebrate models invaluable for studying skeletal biology and its associated disorders. Both teleosts and mammals have key skeletal tissues (bone and cartilage) and cell types (chondroblasts, chondrocytes, osteoblasts, osteocytes, osteoclasts) [12,38]. Teleosts and mammals exhibit many genetically conserved traits, including similarities in skeletal elements, ossification processes, and bone matrix components. Moreover, genetic studies have shown that similarities between human and zebrafish skeletons extend to their underlying molecular mechanisms [39]. Bone formation in zebrafish occurs through a process similar to humans, involving osteoblast differentiation and the deposition of a mineralized matrix. Bone formation begins with mesenchymal stem cells (MSCs), which can undergo two primary ossification processes: intramembranous (direct MSC to osteoblast differentiation) or endochondral (involving a cartilage template) [40] (Figure 1).

Though zebrafish have a simplified initial skeletal pattern, this basic framework is conserved across vertebrates, with adaptations reflecting aquatic or terrestrial habitats [42]. The main components of the zebrafish skeleton are as follows: (i) the craniofacial skeleton, which includes the parietal bones, jaw bones, and opercles (the bones covering the gills) and (ii) the axial skeleton, consisting of the vertebral column, ribs, intermuscular bones, and the unpaired dorsal, anal, and caudal fins [1,43]. Zebrafish reach sexual maturity at approximately 90 days, at which point they have a standard length (SL)—measured from the snout (front of the head) to the last caudal vertebra—of 1.5 to 2.0 cm. Zebrafish continue to grow throughout their lives, with an increase in skeletal volume, reaching a body length of 3 to 4 cm [43]. They typically live for around 3 years but can occasionally reach 5 years old [44]. Zebrafish can regenerate several organs, including the bony caudal fin, which regrows within two weeks post-amputation. Additionally, they absorb drugs from water, making zebrafish ideal for drug screening and testing compounds’ effects on bone regeneration, particularly through standardized caudal fin assays [45,46]. The regeneration process involves the formation of a blastema, where cells proliferate and differentiate through the sequential expression of osteoblast markers [47,48]. It has been demonstrated that two Runx2 paralogs, *runx2a* and *runx2b,* regulate early bone formation alongside *twist* genes that influence craniofacial development and skeletal differentiation [12,49,50,51]. Additionally, zebrafish collagen I closely mirrors the human collagen structure, making zebrafish an effective model for studying bone biology [52]. Importantly, teleost fish possess cycloid scales that contain calcified tissue composed of osteoblasts, osteoclasts, and bone matrix proteins. It is well known that bone remodeling involves a tightly coordinated process in which bone resorption by osteoclasts is closely coupled with bone formation by osteoblasts [53,54,55]. Additionally, signaling molecules involved in cell-to-cell communication, such as receptor activator of nuclear factor kappa-B ligand (RANKL) and osteoprotegerin (OPG), are produced by osteoblasts [56]. In particular, osteoblasts secrete RANKL, which attaches to the RANK receptor on the surface of osteoclasts, promoting bone resorption. Conversely, OPG is released by osteoblasts as a decoy receptor for RANKL, preventing RANKL from binding to RANK, thereby inhibiting bone resorption. Thus, the RANK–RANKL–OPG pathway plays a crucial role in regulating osteoclastogenesis mediated by osteoblasts [57]. In contrast, semaphorin 4D is expressed on osteoclasts, and its binding to the Plexin-B1 receptor on osteoblasts results in the suppression of bone formation [57]. These molecules act as positive and negative regulators of osteoclast development and function, respectively [56,57]. Thus, zebrafish can be used to generate disease models of osteoporosis [58,59]. In humans, recessive loss-of-function mutations in the *LRP5* gene, a co-receptor in the Wnt signaling pathway, lead to osteoporosis-pseudoglioma syndrome [60]. Furthermore, several genome-wide association studies (GWASs) have identified *LRP5* as a key risk locus for osteoporosis-related traits. Recent studies have shown that first-generation (F0) mosaic mutant zebrafish generated by CRISPR-Cas9 mutagenesis, often referred to as “crispants”, can effectively replicate the phenotype of germline knockout (KO) models. The crispant model has been compared with a stable *lrp5* knockout zebrafish model to demonstrate the suitability of crispants for the functional validation of osteoporosis candidate genes [59]. Zebrafish mutants with defects in bone development, such as *runx2b* mutants, exhibit skeletal dysplasia that mimics human bone diseases, increasing the model’s utility for studying osteopathies. Notably, zebrafish have also been instrumental in exploring the role of the genes involved in bone resorption, such as *rankl*, *opg*, and semaphorin 4D, which are essential for the balance between osteoblast and osteoclast activity [12]. These genes are critical in understanding diseases like osteoporosis, where excessive bone resorption occurs. Using a zebrafish model and examining the scales, Kitamura et al. discovered that both dynamic and static acceleration at 3.0 × g led to a reduction in the RANKL/OPG ratio and to an increase in osteoblast-specific functional mRNA, such as alkaline phosphatase. In contrast, static acceleration resulted in an increase, while dynamic acceleration caused a decrease in osteoclast-specific mRNA, like cathepsin K. Additionally, static acceleration enhanced the expression of semaphorin 4D mRNA, whereas dynamic acceleration showed no significant effect. The findings of this study suggest that osteoclasts exert dominant control over bone metabolism through semaphorin 4D expression, which is stimulated by static acceleration at 3.0 × g [61]. This finding indicates that mechanical acceleration influences bone metabolism by modulating the balance between osteoclastogenic and osteoblastogenic signals, thereby promoting osteoblast activity. Furthermore, CRISPR-Cas9 methods have facilitated the creation of heritable disease models in zebrafish, enabling significant insights into the genetic pathways relevant to human health, despite some challenges posed by genome duplication [62]. Numerous zebrafish mutants for osteogenesis imperfecta (OI) have been developed, including the Chi/+ model with a dominant type III OI mutation and the p3h1−/− model mimicking recessive type VIII OI, generated via ENU mutagenesis and CRISPR-Cas9, respectively [63]. Recently, bone structure, cellular and molecular patterns, and matrix assembly were examined in two zebrafish models of osteogenesis imperfecta (OI) using the caudal fin as a model system. This is the first direct comparison of two OI models with different molecular defects: the Chi/+ model, with a collagen defect causing dominant OI, and the p3h1−/− model, with an enzyme deficiency leading to recessive OI [64]. Moreover, Debaenst S. et al. evaluated a semi-high throughput zebrafish platform for the rapid in vivo testing of the candidate genes linked to heritable fragile bone disorders (FBDs) [65]. Using CRISPR-Cas9, six genes related to OI and four associated with bone mineral density were targeted. High indel efficiency (88%) in CRISPR-Cas9-induced F0 mosaic (crispant) zebrafish showed successful gene knockouts, with skeletal characteristics assessed at multiple developmental stages using microscopy, staining, and micro-CT [65]. Larval crispants showed variable osteoblast and mineralization changes, while adults displayed more consistent skeletal abnormalities, including vertebral fractures [65]. Overall, this approach demonstrates that zebrafish crispant screening is a feasible and effective tool for evaluating the genes involved in FBDs. Moreover, due to their predisposition to develop scoliosis and their high genetic similarity to humans, zebrafish are increasingly utilized as a model organism for studying scoliosis in the fields of biomedicine [66]. The use of this model is also supported by the fact that the mechanical forces generated during swimming are applied to the spine in a manner similar to that observed in humans, unlike quadrupedal models such as mice and rats [67,68,69]. Thus, zebrafish scoliosis models have been effectively developed using both N-ethyl-N-nitrosourea (ENU) mutagenesis and reverse genetic techniques. In particular, zebrafish mutant models for genes such as dstyk, col8a1a, tbx6, myadm, col1a1a, col1a2, col1a1b, myhz2, col2a1a, meox1, and stat3 have been developed to resemble congenital scoliosis [66]. The zebrafish model (Table 1) thus provides valuable insights into conserved genetic pathways that may have broader implications for advancing our understanding of bone disorders and enhancing regenerative medicine techniques in vertebrates.

### 2.2. Muscle Disorders

In humans, muscle disorders and myopathies can be broadly divided into inherited and acquired [78,79]. Typically, these disorders present symptoms such as loss of strength, muscle weakness, disability, and in some cases, deformity, depending on the severity and progression of the disease. The diagnostic workup—including clinical evaluation, blood testing, immunohistochemistry, and sometimes muscle biopsy—is mandatory for correct treatment and prognosis [80]. Muscular dystrophies are inherited myogenic disorders that can be divided into more than 30 subgroups [81]. The most common muscular dystrophy is Duchenne muscular dystrophy (DMD), occurring in approximately 1 in every 5000 male births [82]. This progressive muscle alteration is characterized by delayed motor development in infancy, leading to the loss of mobility by the end of the first decade, and ultimately causing respiratory or cardiac failure in early adulthood. DMD is caused by mutations in the dystrophin gene, located on chromosome Xp21 [83]. Dystrophin plays a major role in maintaining fiber integrity by transmitting muscle force and protecting the sarcolemma from mechanical stress. Additionally, dystrophin regulates muscle satellite stem cell division [84,85]. Indeed, patients with DMD have striated muscles that lack the rod-like protein, dystrophin, which is due to inherited or spontaneous mutations in the X-linked dystrophin gene [86]. In skeletal muscles, dystrophin is found at the inner surface of the sarcolemma, where its NH2 terminus binds to actin, while its COOH terminus connects to the transmembrane protein, β-dystroglycan [86]. Dystrophin plays a crucial role in stabilizing the cell membrane against the mechanical forces generated during muscle contraction [86]. DMD and other forms of muscle degeneration can be modeled in zebrafish by targeting specific muscle-related genes. In particular, among preclinical disease model organisms such as *Drosophila melanogaster*, *Caenorhabditis elegans*, or zebrafish, only zebrafish share the fundamental morphology, physiology, and genomics found in all vertebrates. The zebrafish dystrophin orthologue encodes a protein with a high degree of homology to human dystrophin at both the NH2 and COOH termini [87]. In 2003, Bassett et al. demonstrated that zebrafish mutants (“sapje”) could be used to investigate the pathogenesis of human muscular dystrophy [88]. The study highlighted that sapje mutants exhibited a phenotype due to the failure of embryonic muscle end attachments. Specifically, sapje mutant larvae exhibit muscle degeneration as early as 3 days post-fertilization [89]. Thus, dystrophic zebrafish strains, such as sapje and sapje-like, have been employed to screen over 1000 FDA-approved drugs and bioactive compounds, leading to the identification of several promising therapeutic candidates for DMD [87,88,90,91,92]. Interestingly, Widrick et al. conducted a thorough evaluation of the validity of using 4–7 days post-fertilization (dpf) sapje larvae as a model for the muscle dysfunction seen in DMD [93]. Zebrafish mutants with locomotor defects can be used as valuable models for identifying the potential therapeutic implications for human diseases, since zebrafish muscles show contractile and metabolic properties that are remarkably similar to those of human skeletal muscle [94]. Neuromuscular electrical stimulation (NMES), particularly endurance stimulation, has beneficial effects on muscle structure and function in zebrafish, suggesting that endurance physical activity may be valuable in DMD patients [95]. Mutant zebrafish are also used as models in relation to other muscular diseases, such as Becker muscular dystrophy [87,96], congenital muscular dystrophy caused by mutations in the human *laminin* α*2* (*LAMA2*) gene [97], X-linked myotubular myopathy [98], Centronuclear Myopathies [99], and Myotonic Dystrophy Type I [100,101], among others [102]. These studies demonstrate that *Danio rerio* is not only a feasible model for studying muscular diseases, but its use is also a relatively easy way to monitor live systems for real-time observation [103].

The zebrafish model (Table 2) can be instrumental in finding the underlying causes of acquired myopathies and potentially preventing them. Muscular disorders can be caused by the iatrogenic effects of medications (i.e., statin-induced myopathy) [104]. Some studies have shown changes in muscle cytoskeleton structure in zebrafish embryos, including the dysregulation of PPAR gene expression and changes in the extracellular matrix when exposed to statins [105,106]. Similarly, exposure to fibrates has been shown to result in alterations in muscular fibers and neuromuscular junctions (119). Furthermore, the drug, Ezetimibe, seems to lower creatine kinase levels in zebrafish larvae [107]. However, differential diagnosis between acquired myopathies, late-onset genetic myopathies, and age-related sarcopenia may be challenging [108].

In this field, zebrafish models can be considered useful tools in biomedical research and, in particular, in muscle diseases such as sarcopenia [120]. Researchers developed Electrical Impedance Myography (EIM) to study the muscle atrophy in *Danio rerio* models, a method that is also used in humans, with a robust correlation to histological-based morphometric analysis [121]. Additionally, Rutkove et al. suggest that EIM in zebrafish may be a new feasible tool to study other skeletal neuromuscular disorders [122]. Zebrafish can serve as a model for secondary sarcopenia, such as vitamin E deficiency-related muscle wasting. In fact, it has been highlighted that chronic exposure to low vitamin E levels leads to oxidative damage that is associated with amino acid and purine metabolic pathway dysfunction in 55-day-old zebrafish [123]. A study by Yun-Yi et al. focused on sarcopenic obese zebrafish (SOB), induced by a high-fat diet. A higher expression of the atrophy-related markers, Atrogin-1 and muscle RING-finger protein-1, was found in SOB zebrafish, with a significant decrease in the skeletal muscle mass and exercise capacity [124]. On the other hand, exercise interventions have been shown to counteract sarcopenia, not only in humans but also in zebrafish models, further confirming their utility in translational medicine. For instance, aerobic exercise has been found to improve the muscle function and quality in D-galactose-induced sarcopenic zebrafish by modulating the miR-128/IGF-1 pathway and enhancing mitochondrial homeostasis [125]. Additionally, a study found that 8 weeks of exercise helped counteract sarcopenia by improving the key cellular processes. Specifically, the exercise restored balance in the AMPK/SIRT1/PGC-1α pathway (important for energy production), reduced the levels of the inflammatory enzyme, 15-PGDH, and boosted the formation of new mitochondria, which are crucial for muscle energy and health [126].

## 3. Muscle–Bone Crosstalk

**Figure 2:**
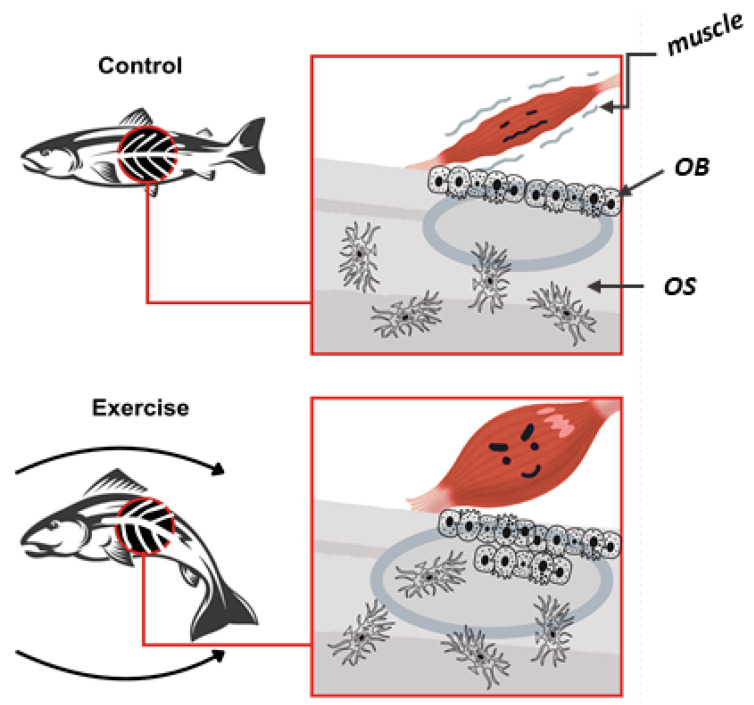
Effects of exercise on zebrafish bone quality: The exercised zebrafish (bottom) shows greater muscular forces, increasing bone load. This stimulated osteoblast activity, enhancing bone formation and mineralization. Consequently, exercised zebrafish had greater bone mass and higher mineralization compared to controls. OB (osteoblasts); OS (osteocytes).

Muscle and bone are two distinct yet closely interconnected tissues that work together to allow movement as well as to maintain structural integrity and homeostasis throughout the body. The communication between these tissues, known as muscle–bone crosstalk, is essential during both the developmental stages and throughout life, influencing growth, repair, and adaptation in response to various stimuli such as physical activity, injury, or disease [127]. This crosstalk is mediated by a complex interplay of biochemical signaling molecules, mechanical forces, and metabolic pathways, making it crucial for the maintenance of overall musculoskeletal health [127]. During development, muscle and bone need to grow and adapt in a coordinated manner to ensure proper biomechanical function. Bone provides a scaffold that supports the attachment of muscles, while muscle activity generates the mechanical forces necessary for bone formation and remodeling [128,129,130]. In addition, mechanical forces, such as those generated during muscle contractions, play a critical role in shaping bone architecture by stimulating osteoblasts [131,132,133]. This process, known as mechanotransduction, is essential for bone strength, density, and overall skeletal integrity. Mechanical loading, which refers to the physical forces exerted on tissues during activities such as movement, muscle contraction, and weight bearing, influences the musculoskeletal health in zebrafish, as it does in other vertebrates [134,135,136]. In zebrafish, these forces are primarily generated during swimming, a natural form of locomotion involving constant muscle contraction and relaxation. This activity produces mechanical stimuli that are essential for the normal development, maintenance, and homeostasis of both muscle and bone tissues [137,138]. Studies in zebrafish have shown that swimming activity directly influences the growth and structural integrity of bones [139]. When zebrafish engage in regular swimming, the mechanical forces produced by muscle contractions are transmitted to the bones through the tendons, triggering a cascade of molecular signals within bone cells, such as osteoblasts [33,140]. Mechanotransduction pathways allow bones to sense mechanical stimuli and convert them into biochemical signals, which then activate the genes and pathways responsible for bone formation and strength [141]. The mechanical forces generated by muscle activity induce stress on bones, which respond by reinforcing their structure to accommodate the increased demand [128,135]. One of the key outcomes of this process is the regulation of bone density and architecture, ensuring that bones adapt to the mechanical demands placed on them by muscle activity [14,135]. It has been demonstrated that zebrafish subjected to exercise show an increased bone mass and a greater degree of bone mineralization compared to the control group [139] (Figure 2).

In humans, enhanced physical activity and higher levels of bone mineralization are also positively associated with improved bone quality [142]. The findings from these zebrafish exercise experiments offer valuable insights into the intricate mechanisms underlying the mechanical sensitivity of bone, as well as the pathways involved in bone formation and mineralization, which ultimately influence bone quality. Conversely, when the mechanical loading is reduced—such as when zebrafish exhibit muscle dysfunction or impaired swimming ability due to genetic mutations or experimental conditions—bone development becomes significantly altered [139]. Reduced or absent muscle contractions lead to a lack of the mechanical stimuli that are critical for healthy bone formation. This results in impaired bone growth, reduced bone mass, and changes in bone architecture, which resemble conditions like osteopenia or osteoporosis in humans [135,143,144]. These findings highlight the mechanical aspect of muscle–bone crosstalk, where a decrease in muscle activity directly impacts bone health, demonstrating the interdependence of these tissues. This mechanical crosstalk is not limited to bone development but also plays a fundamental role in muscle growth and maintenance. In turn, bones provide the structural framework that muscles rely on to generate force and movement, emphasizing the bidirectional relationship between these systems in maintaining overall musculoskeletal health [145,146]. This reciprocal relationship ensures that both tissues develop in harmony and adapt to changing physical demands over time. In zebrafish, these mechanical forces are particularly crucial during early development, when swimming behavior begins as soon as the larvae develop functional muscles and the skeletal system [137,147,148]. This early mechanical loading is a pivotal factor behind the proper maturation of the musculoskeletal system. Even a few disruptions in swimming activity during these critical stages of development can lead to long-term alterations in both muscle strength and bone structure. For example, mutations in genes that affect muscle function, such as those causing muscular dystrophy in zebrafish models, not only lead to muscle weakness but also induce negative effects on bone health, providing clear evidence of the mechanical link between the two systems [149]. Moreover, zebrafish models have been used to explore how specific molecular pathways mediate mechanotransduction in bone [150,151]. In addition, the myotendinous junction (MTJ) plays a key role in muscle–bone interaction by enabling force transmission during muscle contraction [152,153]. Recent advancements in MTJ research have highlighted its molecular and structural components, essential for tissue integrity [154,155]. Given its potential in studying musculoskeletal diseases, zebrafish models, with their genetic tractability and developmental transparency, are ideal for investigating the MTJ and muscle–bone interactions. Using the zebrafish model, the dynamic and complex spatial-temporal nature of MTJ morphogenesis has been highlighted. Specifically, the attachment of fast-twitch myofibers to the MTJ is linked to the formation of novel microenvironments [156]. This process involves the upregulation of focal adhesion kinase (Fak) and β-dystroglycan, alongside the downregulation of the extracellular matrix protein, fibronectin (Fn). The degradation of Fn creates a distinct microenvironment near slow-twitch fibers but not fast-twitch ones. Interestingly, Fak, laminin, Fn, and β-dystroglycan also accumulate at the MTJ in mutants lacking slow-twitch fibers [156]. In addition, deleting the MTJ marker gene, col22a1, in zebrafish has been shown to result in MTJ dysfunction with varying severity and distinct phenotypes. Most individuals survive to adulthood without obvious muscle defects, while others display severe motor impairments and die before metamorphosis. Despite this, all mutants exhibit muscle weakness caused by inefficient force transmission, which disrupts locomotion-related functions. Thus, this study positions COL22A1 as a candidate gene for the myopathies linked to defective force transmission and emphasizes the phenotypic heterogeneity of the condition [157].

Muscle atrophy or dysfunction, which results in decreased mechanical loading, can lead to conditions such as osteopenia or osteoporosis, where the bone mass and strength are reduced [21,158]. On the biochemical level, muscle and bone communicate via signaling molecules known as myokines (produced by muscles) and osteokines (produced by bones) [15,22]. These molecules act as mediators in muscle–bone crosstalk, regulating various cellular processes in both tissues [16]. For instance, myokines like myostatin play a key role in inhibiting muscle growth and have been shown to negatively impact bone formation as well [15,127]. Conversely, osteocalcin, a hormone secreted by osteoblasts in bones, has been implicated in regulating muscle function and metabolism [159,160]. The maintenance of muscle mass depends on the balance between anabolic (protein synthesis) and catabolic (muscle breakdown) events. Mera et al. found that osteocalcin signaling in muscle fibers promotes protein synthesis [161]. Other anabolic hormones activate the PI3K/Akt/mTOR pathway to stimulate protein synthesis and muscle hypertrophy [162]. Thus, osteocalcin is necessary and sufficient to stimulate Akt phosphorylation in muscle during exercise, promoting protein synthesis in mouse myotubes through the activation of the mTOR pathway [161]. The balance and regulation of these signaling pathways are essential for coordinating muscle and bone growth and repair, especially during periods of physical activity or following injury [163].

Zebrafish may provide an excellent model system for studying muscle–bone crosstalk. They are amenable to genetic manipulation, enabling scientists to create models of musculoskeletal diseases by mutating or knocking out the specific genes involved in muscle or bone functions [164,165,166]. Different levels and timing of Hedgehog (Hh) signaling specify three different cell types in the zebrafish myotome. Two of these cell types, the medial fast-twitch fibers (MFFs) and the slow-twitch muscle pioneers (MPs), are defined by the expression of the genes, *eng1a*, *eng1b*, and *eng2a*, and depend on the highest levels of Hh signaling for their maturation. Indeed, Maurya et al. identified in the zebrafish myotome a minimal *eng2a* element sufficient to drive the reporter expression specifically in MPs and MFFs by binding both Gli2a, a key Hh signaling mediator, and pSmads, which are activated by bone morphogenetic protein (BMP) signaling [167]. In addition, the authors demonstrated a negative correlation between pSmad accumulation in the nucleus and *eng2a* expression. They found that the nuclear accumulation of pSmad negatively affects the expression of *eng*, while preventing this accumulation allows for the activation of *eng2a*, regardless of the level of Hh signaling. Additionally, maximal Hh signaling depletes nuclear pSmads [167]. Therefore, these findings suggest that the *eng2a* promoter integrates both repressive BMP signals and activates Hh signals to restrict the gene expression to MPs and MFFs. In addition, this study suggests a novel interaction between Smads and truncated Gli proteins, highlighting a new mechanism of crosstalk between the BMP and Hh pathways.

Moreover, zebrafish have the ability to regenerate both muscle and bone tissues after injury, providing further insights into the molecular mechanisms that drive the repair processes in these tissues [168]. Mammals typically have a limited ability to regenerate, mainly confined to specific organs such as the liver and fetal skin [169,170]. In contrast, other vertebrates such as salamanders, lizards, and teleost fish possess the ability to regenerate a wide range of body parts [171]. The regenerative capacity of the musculoskeletal system differs among vertebrates; for example, humans can repair muscle and bone after injury and recover full function, as long as there is no significant loss of tissue [168]. Studying the processes of regeneration in various vertebrates is key to understanding which traits have evolved or been lost over time. Furthermore, zebrafish models of clinically relevant muscle and skeletal injuries mirror mammalian regeneration. Indeed, following muscle damage, zebrafish quiescent stem cells, called satellite cells, are activated, and proliferate, differentiate, and fuse to create new myofibers [168]. In the case of bone fractures, the healing process occurs in stages, starting with hematoma formation and inflammation, fibrocartilage callus formation, bony callus development, and finally remodeling [172,173]. These zebrafish models are ideal for testing gene therapies or pharmacological treatments aimed at addressing conditions such as muscle tears and fractures.

## 4. Aging and the Musculoskeletal System

Life expectancies are rising significantly, and by 2030, one in six people globally will be 60 years or older. By 2050, the number of people aged 60 and above will have doubled, while the population aged 80 and over will have tripled. The association between telomeres and aging is underscored by the fact that genetic disorders leading to telomerase deficiency are linked to premature aging [174]. This relationship has primarily been studied in mice, which have long telomeres [175,176]. However, zebrafish have recently emerged as a powerful and complementary model for exploring telomere biology. They exhibit human-like short telomeres that progressively shorten with age [177]. The comprehensive characterization of their well-conserved molecular and cellular physiology makes zebrafish an excellent model for unraveling the complex relationship between telomere shortening, tissue regeneration, aging, and disease [177]. Studies have shown that telomeres in the muscles of wildtype zebrafish shorten with age, eventually reaching the length typical of mutants lacking telomerase, an enzyme that normally helps maintain telomere length [178]. In addition, telomere shortening in the gut and muscles of zebrafish has been shown to drive the systemic aging process [178].

Aging is a key risk factor for many diseases, including sarcopenia, a condition marked by the gradual loss of skeletal muscle mass, strength, and function, which results in functional decline, frailty, and a higher risk of mortality [179]. As a result, an increase in cases of sarcopenia and related deaths is anticipated in the coming years. While the precise mechanisms underlying sarcopenia are not yet fully understood, the condition is known to involve changes in muscle structure and function, diminished regenerative capacity, oxidative stress, inflammation, and mitochondrial dysfunction [180]. Therefore, utilizing enhanced and diverse animal models is crucial for achieving a thorough understanding of sarcopenia’s pathogenesis. Although rodents are the most commonly used models for sarcopenia research, alternative models like Drosophila and *C. elegans* have been applied due to their lower cost and shorter lifespans. However, it is important to recognize that these models differ significantly from human skeletal muscle [181]. In this context, the zebrafish presents itself as a novel and promising model to explore. The zebrafish has been recognized as a versatile animal model for the study of aging-related diseases [182]. A convenient aspect that makes the zebrafish a valuable model organism for aging research is its relatively long lifespan (a maximum of at least five years), which increases its representativeness of the lifespan of mammals [44]. Furthermore, zebrafish models have relatively low costs of maintenance, are amenable to genetic manipulation, and have high growth rates, which facilitate the rapid obtainment of large populations for demographic studies [44]. Interestingly, *Danio rerio* is a fundamental model to study the physiopathological consequences of aging like telomere malfunction [183].

In addition, hundreds of milligrams of skeletal muscle can be obtained from a single zebrafish individual [44]. The zebrafish has been used to study frailty and sarcopenia, revealing the aging-related alterations in the growth and differentiation of skeletal muscle [94,120,149,184,185]. Age-related sarcopenia is a muscle disorder linked to an imbalance between protein synthesis and breakdown, but also to impaired mitochondrial permeability and biogenesis [126]. Due to their swimming abilities, zebrafish are a good model to test the effects of aerobic exercise on skeletal muscle fitness in sarcopenic animals [125]. They can also be used for the electrophysiology measurements of the ion channels involved in muscle excitability [186] and to study skeletal muscle senescence [187]. The zebrafish is an emerging animal model of osteoarthritis [188]. The zebrafish has been used to study the impact of Efemp1 (EGF-containing fibulin extracellular matrix protein 1, a protein of the extracellular matrix that is upregulated in the blood, urine, and bones of osteoarthritic patients) in osteoarthritis [189]. As senescence plays a key role in the development and progression of various aging-related diseases and frailty, there has been growing interest in mechanistic research and the search for compounds that target senescent cells, known as senolytics [190]. Mammalian models are typically used to test senolytics and gather functional and toxicity data at the organ and system level, but these approaches are costly and time consuming. Zebrafish, which share significant genetic homology with humans in genes linked to aging and disease, offer an alternative model that can be genetically modified with relative ease. A transgenic zebrafish line expressing the senescence marker, p21, fused to GFP has been generated. In these animals, the number of cells with p21-GFP fluorescence increases with natural aging and upon exposure to ionizing radiation, an effect that was decreased with senolytics [191].

## 5. Zebrafish and Therapeutic Strategies

Zebrafish have become extensively used for exploring the potential new therapeutic approaches for both common and rare bone and muscle diseases, including muscular dystrophy, osteoporosis, and osteogenesis imperfecta (OI) [192]. Zebrafish larvae can absorb molecules through their mouth and gills, enabling the screening of compounds dissolved in the water to treat multiple samples. Malformations, such as embryo coagulation, the absence of somite formation, non-detachment of the tail, and lack of a heartbeat, can be quickly identified using bright-field microscopy [193]. More recently, new and faster methods for drug screening have been developed, utilizing juvenile and adult zebrafish. By the time that juveniles reach 1 cm in length, they have acquired most adult morphological and physiological features and can still be housed in 24-well plates, which require small volumes, thus enabling cost-effective and reasonably high-throughput screening. This method has been used to investigate the role of NF-κB signaling during osteoblast dedifferentiation and has revealed an unexpected function of NF-kB signaling in maintaining the differentiated state of osteoblasts [194]. Zebrafish are able to regenerate the skeletal tissues after amputation of the tail fin or removal of elasmoid scales [195]. Since the fins and scales are translucent and easily imaged, they allow the detailed visualization of cells and their calcified matrix using standard fluorescent microscopes. Thus, the process of fracture repair can be monitored in real time at a cellular level using transgenic lines or by labeling bone formation with tetracycline and Calcein to perform histomorphometric analysis [196,197]. Another study employed a semi-automated imaging strategy using Calcein-stained larvae exposed to a small-compound library, which led to the identification of six catabolic and two anabolic compounds that affect notochord mineralization [198]. Fin regeneration assays have proven effective for evaluating bioactive compounds, as shown by experiments using regenerating fins treated with glucocorticoids. This treatment led to a reduction in bone formation, along with a decrease in both the number of osteoblasts and subsequent bone deposition, as well as reduced osteoclast recruitment in these fins [199]. Zebrafish models of osteoporosis have been used to screen for drugs that enhance osteoblast activity or inhibit osteoclast-mediated bone resorption. According to what we observe in mammals, glucocorticoid-induced osteoporosis increases the risk of fracture. Moreover, zebrafish vertebrae in a glucocorticoid-induced osteoporosis (GIOP) model were used to test prednisolone-reduced bone mineral density (BMD), while alendronate alone improved bone hardness and elasticity. Sequential treatment with both drugs restored BMD to healthy levels and maintained the vertebral structure. These results suggest that alendronate, particularly when administered after prednisolone, effectively counteracts GIOP-related bone deterioration in zebrafish [200]. Wang et al. investigated the role of adenosine in osteoporosis and oxidative stress using zebrafish models, demonstrating its capacity to enhance cell proliferation and increase alkaline phosphatase activity. The PI3K/Akt pathway was identified as the key mechanism mediating adenosine’s effects, highlighting its potential as a therapeutic agent for osteoporosis management [201]. Using the zebrafish model, it has been demonstrated that pinoresinol treatment resulted in enhanced bone mineralization and corrected cartilage malformations and spinal curvature, improving swimming abilities [202]. Zebrafish have been used to test new osteo-active compounds in osteoporosis [58] and also for the CRISPR-based screening of the genes potentially involved in osteoporosis and the screening of osteogenic compounds [59]. Small-molecule screens in zebrafish have identified compounds that promote bone formation or prevent muscle degeneration. Indeed, zebrafish have been used to test bone regeneration induced with hydroxyapatite nanoparticles [203]. Further studies have evaluated the potential of chito-oligosaccharides (COS) to stimulate osteoblast differentiation and offer protection against osteoporosis, using both mouse MSCs and zebrafish models. In zebrafish, COS supports bone repair and mineralization, mitigates osteoclastic activity, and improves the calcium-to-phosphorus ratio, demonstrating protective effects in dexamethasone-induced osteoporosis. These findings suggest that COS may act through the MMP3–Osteopontin–MAPK signaling pathway, highlighting its potential as an osteoporosis treatment [204,205,206]. Therefore, the use of zebrafish as models for testing the toxicity of osteoporosis drugs is widely adopted [42,207,208]. Recently, a study highlighted and formulated zein nano coop composites containing chimeric antioxidants-ascorbic acid, luteolin, resveratrol, and coenzyme Q (AZN), as a promising treatment for osteoporosis, demonstrating its ability to promote bone regeneration without toxicity across various stages of zebrafish development [209]. AZN treatment in zebrafish larvae improves bone formation, enhances calcium and phosphorus deposition, and reduces osteoclast activity, indicating its potential to counteract osteoporosis. These findings highlight AZN’s therapeutic promise for bone health and support the need for further research into its clinical applications [209]. Recent advancements in human genomic and transcriptomic data have identified potential osteo-anabolic factors. A new screening pipeline using genetically tractable zebrafish offers a cost-effective, high-throughput alternative to the traditional models [58]. After identifying the candidate genes and drug targets from human genetic studies, the pipeline involves two experimental approaches that can be conducted concurrently to generate pre-clinical data validating these targets [58]. Genome editing, such as CRISPR-Cas9, allows for loss-of-function studies in transgenic zebrafish to assess the impact of specific genes on skeletal development and mineralization, while also testing for potential adverse effects on other tissues or organs. This method enables the creation of hundreds of mosaic zebrafish mutants in just 3–4 weeks, which is challenging to achieve with other systems like cultured chondrocytes or osteoblasts [58]. Furthermore, CRISPR-Cas9 enables the study of specific human disease mutations in zebrafish, as long as they occur in conserved coding regions [210,211]. These zebrafish can develop into adults, with germline mutations identified, allowing for more in-depth studies of the mature skeleton.

Similarly, muscle dystrophy models have been employed to test compounds that improve muscle strength and function. Phosphodiesterase (PDE) inhibitors are compounds that have shown promise in improving the muscle function in dystrophin-deficient zebrafish [109]. Aminophylline, a nonspecific PDE inhibitor, and sildenafil citrate, a PDE5 inhibitor, were among six compounds that improved the muscle morphology, increased the vascularization, and prolonged the survival of dystrophin-null larvae [212]. The positive effects of aminophylline on rescuing dystrophin-deficient sapje larvae were later confirmed by an independent group using an unbiased screen of the ENZO FDA Approved Drug library [92]. To further explore this class of drugs, five additional PDE inhibitors were tested in dystrophic zebrafish. Among them, ibudilast, rolipram, and dipyridamole improved the dystrophic phenotype in 4-day-old sapje larvae, though they were not as effective as aminophylline or sildenafil [91]. In contrast, the PDE3 inhibitors, enoximone and milrinone, showed no therapeutic benefit [91]. Moreover, targeting muscle–bone crosstalk offers a novel therapeutic approach. By focusing on pathways that simultaneously affect both muscle and bone health, such as myostatin inhibitors or WNT activators, it may be possible to develop therapies that address both muscle and bone degeneration in diseases like osteoporosis and muscular dystrophy.

## 6. Limitations of the Zebrafish Model

While the zebrafish model offers numerous advantages for studying musculoskeletal disorders, it is essential to acknowledge its inherent limitations to provide a balanced and comprehensive perspective. One significant limitation arises from the fundamental differences in environmental pressures between terrestrial and aquatic life. Teleosts experience gravity differently than terrestrial vertebrates due to the higher density of water compared to air, which reduces its direct effects [213]. Instead, gravity acts indirectly through hydrostatic pressure [213]. Consequently, the teleost skeleton primarily adapts to the mechanical forces from movement (e.g., swimming, feeding) rather than the gravitational load [213]. However, gravity can be manipulated experimentally to study its impact on the teleost bone structure [214]. The physicochemical characteristics of laboratory water conditions may vary significantly from those in natural environments [2]. In particular, laboratory environments do not replicate the constant gravitational and mechanical forces experienced in terrestrial conditions. This discrepancy may affect the translational applicability of zebrafish findings to human musculoskeletal disorders, where such forces play a critical role in the development and function of bones and muscles. Additionally, the specific biological differences between zebrafish and humans can present challenges. Zebrafish myogenesis displays unique characteristics, highlighted by the unexpected distributions of the key muscle cytoskeletal proteins, such as actin, myosin, desmin, α-actinin, troponin, and titin, which contribute to the distinct aspects of muscle formation and organization in this model [215]. Furthermore, in the context of muscular dystrophies, the zebrafish model lacks the utrophin gene, which plays a compensatory role in humans [216]. As a result, zebrafish with Duchenne muscular dystrophy exhibit a more rapid disease progression. While this accelerated timeline can be advantageous for experimental purposes, it may not fully capture the complexities of disease progression in humans. Thus, recognizing and addressing these challenges will enhance the utility of zebrafish as a complementary model in translational research. In addition, when selecting an animal model, it is crucial to adhere to general guidelines to ensure reliable and reproducible results. This principle is particularly relevant for zebrafish, where specific considerations must be addressed. The presence of gene polyploidy has been documented and requires careful verification. The zebrafish genome underwent an additional duplication event after the divergence of the fish and mammalian lineages, resulting in cases where zebrafish can exhibit polyploidy for certain genes [217,218]. Numerous zebrafish mutants have been generated and genetically isolated through the traditional diploid genetic screens. This process suggests that many of the duplicated genes have either lost their functionality or have undergone subfunctionalization [219]. Importantly, as with all animal models, the genetic background must be homogenized through crossbreeding to minimize the variability. The significant genetic variability both between and within zebrafish strains can influence phenotypes, potentially affecting the experimental outcomes. If not carefully managed, this high level of germ-line variation and population substructure in this widely used model organism could introduce confounding factors, complicating the efforts to translate the findings to human diseases [220]. Furthermore, the cell type lineage under investigation may not be directly comparable to that in other models or systems, so it is essential to thoroughly identify the cell lineage involved in the study to ensure accurate and interpretable results, avoiding the potential errors caused by the differences in cell types across different models [221].

## 7. Conclusions

Despite these limitations and the need for careful evaluation of the model used, zebrafish have proven to be invaluable for advancing our understanding of the developmental processes underlying musculoskeletal disorders. Their genetic tractability, coupled with the ability to visualize dynamic processes in vivo, makes zebrafish an ideal model for studying the intricate interplay between muscle and bone. The muscle–bone crosstalk observed in zebrafish mirrors that of humans, as many of the molecular pathways and mechanisms regulating these interactions are conserved across species. This similarity allows zebrafish to serve as a powerful platform for identifying the potential therapeutic targets and testing new treatments in a cost-effective and efficient manner. By studying zebrafish, researchers can rapidly evaluate the impact of genetic modifications or drug compounds on musculoskeletal systems, bridging the gap between basic research and clinical applications. Future research leveraging the zebrafish system will likely uncover additional pathways involved in muscle–bone communication, offering new therapeutic opportunities to benefit patients suffering from musculoskeletal disorders.

## Figures and Tables

**Table 1:** Selected zebrafish models and their applications in bone diseases and disorders.

Zebrafish Model	Applications	Ref
LRP5 mutant or crispant	Osteoporosis	[59]
runx2b mutants	Bone development	[70]
Chihuahua (Chi/+)	Osteogenesis imperfecta	[63,64]
p3h1−/− model	Osteogenesis imperfecta	[63,70]
CRTAP mutant	Osteogenesis imperfecta	[71]
fkbp10a mutant	Bone fragility	[72]
aldh7a1, daam2, esr1 and sost crispant	Osteoporosis	[65]
creb3l1 ifitm5 mbtps2 sec24d serpinf1 and sparc crispant	Osteogenesis imperfecta	[65]
dstyk mutant	Scoliosis	[73]
col8a1a mutant	Scoliosis	[74]
tbx6 mutant	Scoliosis	[75]
myadm mutant	Scoliosis	[76]
col2a1a mutant	Scoliosis	[76]
stat3 mutant	Scoliosis	[77]

**Table 2:** Selected zebrafish models and their applications in muscle diseases and disorders.

Zebrafish Model	Applications	Ref
sapje	Duchenne muscular dystrophy	[88,89,90,93,109]
sapje-like	Duchenne muscular dystrophy	[87]
candyfloss (lama2 mutant)	Congenital muscular dystrophy	[97]
Tg(ACTA1 D286G-eGFP)	Nemaline myopathy	[110]
*softy (lamb2mutant)*	Muscular dystrophy lamb2 mutation	[111]
mtm1 morpholin	Myotubular myopathy	[98,112]
*dmd morpholin*	Duchenne muscular dystrophy	[113]
*col6a1 morpholin*	Ullrich congenital muscular dystrophy; Bethlem myopathy	[114]
*dysf morpholino*	Myoshi myopathy; limb–girdle muscular dystrophy	[91,115]
*fkrp morpholino*	Multiple forms of dystroglycanopathy	[116,117,118]
ryr1b mutant	Multiple forms of congenital myopathy	[119]

## Data Availability

No new data were created.
